# PTSD prevalence among resident mothers and their offspring in Rwanda 25 years after the 1994 genocide against the Tutsi

**DOI:** 10.1186/s40359-019-0362-4

**Published:** 2019-12-19

**Authors:** Celestin Mutuyimana, Vincent Sezibera, Epaphrodite Nsabimana, Lambert Mugabo, Cindi Cassady, Clarisse Musanabaganwa, Yvonne Kayiteshonga

**Affiliations:** 1 Centre for Mental Health, University of Rwanda, Kigali, Rwanda; 2 Hope and Homes for Children, Salisbury, UK; 3 Department of Clinical Psychology, University of Kibungo, Kibungo, Rwanda; 4 Medical Research Centre, Rwanda Biomedical Centre, Ministry of Health, Kigali, Rwanda; 5 Mental Health Division, Rwanda Biomedical Centre, Ministry of Health, Kigali, Rwanda

**Keywords:** PTSD, Prevalence, Survivors, In country non-targeted, Old returnees, Offspring

## Abstract

**Background:**

The 1994 Genocide against the Tutsi was a major traumatic event affecting nearly all Rwandans. Significant psychological sequels continue to occur in the population 25 years after, with a high prevalence of posttraumatic stress disorder (PTSD) found in women. Three groups are typically designated with regard to the Genocide against the Tutsi: those who were targeted and categorized as genocide “survivors,” those who were in the country during the genocide and were the “non-targeted” group, and those who were outside of the country, referred to as the “1959 returnees.” Each group experienced various traumatic events during and in the aftermath of the genocide. Offspring of the designated groups, currently exhibit symptoms of PTSD disregarding of being born in the years following the genocide. A number of studies have described the prevalence of PTSD in the general adult population. There is a lack of research comparing the prevalence of PTSD in women and their offspring among these three target groups, therefore, this study aimed to bridge the gap.

**Methods:**

We conducted a comparative cross-sectional study with a sample of 432 mothers and 432 children in three categories: genocide survivors, in country non-targeted and 1959 returnees. Participant ages for children were between 14 to 22 years and for mothers, between the ages of 32 to 87 years. The UCLA-PTSD DSM-5, PTSD Check list-5 and Life events Checklist-5 were translated from English to Kinyarwanda and were used to assess exposure to trauma and the prevalence of PTSD symptoms in Rwandan mothers and their offspring.

**Results:**

Key Results yield a PTSD rate of 18.8, 6.2, 5.2% within survivors, in country non-targeted, and returnees respectively with an average PTSD rate of 43.8% for parents, and 16.5% for offspring.

**Conclusion:**

PTSD among the mothers’ groups and their offspring have been found, specifically in the offspring of genocide survivors. Considering these adolescents were not born at the time of the 1994 Genocide against the Tutsi, the results suggest future studies should explore the precipitating factors contributing to the PTSD symptoms within this specific group.

## Background

Post-traumatic stress disorder (PTSD), a trauma and stressor related disorder is found worldwide [1]. Estimates of the lifetime prevalence of PTSD vary considerably according to social background and country of residence, ranging from 1.3 to 12.2 [2]. In Europe, differences in PTSD prevalence range from 0.56 to 6.67% in the general population, with one outlier country, Croatia, reporting a ten-fold higher prevalence [3]. In South Africa the risk of developing PTSD after exposure to trauma and the probability of symptom chronicity after the initial onset of PTSD is highest in those who witnessed a traumatic event [4]. Post-traumatic stress disorder is present not only in adults but also in children and is associated with many factors. The prevalence of PTSD is typically elevated in populations with high rates of direct exposure to traumatic events. PTSD in children and adolescents may be caused by either direct exposure to traumatic life events or by generational transmission of trauma [5, 6].

Gender differences have been noted with women developing PTSD two to three times more often than men.

On a global scale, the lifetime prevalence of PTSD is about 10–12% in women and 5–6% men. When mothers are suffering from PTSD, their offspring may also be exposed. In Rwanda, considerable research has focused on the prevalence of PTSD within the general population [7]. With regard to the 1994 Genocide against the Tutsi exposure categories, there were those who were victims; currently categorized as “survivors,” those who were in-country and non-targeted and those who were outside of the country; referred to as the “1959 returnees.”

Less is known about differences in the prevalence of PTSD within specific groups such as women and their offspring. Our objective was to address the following questions:

1. What is the prevalence of PTSD among mothers and their offspring residing in Rwanda 25 years after the 1994 Genocide against the Tutsi? 2. Is the prevalence of PTSD associated with generational groups (mothers versus offspring)? 3. Is the prevalence of PTSD among mothers and their offspring associated with the mothers’ survival category (genocide survivor, returnee, in-country non-targeted)? 4. Is the prevalence of PTSD among offspring associated with their mothers’ survival status and level of exposure to trauma?) We aimed to point out the general prevalence rate from a large sample of dyads of mothers and offspring, and how the survival status in the 1994 Genocide against Tutsi can influence PTSD prevalence not only in mothers but also in offspring. It is hoped this research will open avenues for early identification of PTSD in the offspring groups and create opportunities to develop preventive interventions.

## Methods

### Participants

Participants were selected and placed into one of three groups based on their exposure to the 1994 Genocide against the Tutsi. The groups are representative of the different experiences of Rwandans during the genocide.

The first group was made up of 1994 Genocide against the Tutsi survivors, the second group consisted of in- country non-targeted mothers and the third group, “1959 returnees” was comprised of mothers who lived outside the country during the genocide and who later returned to Rwanda. Each group consisted of dyads of a mother and one of her offspring between the ages of 14 and 22 years old. Study participants came from seven districts: Ruhango, Gasabo, Gisagara, Huye, Rutsiro, Gatsibo,and Kamonyi. The districts were selected on the basis of having large populations of survivors and those who lived outside the country during the 1994 Genocide against the Tutsi. The population size was determined based on the G-power rule of at least 400 participants according to the type of analysis. Our sample was comprised of 432 dyads of mothers and offspring categorized by the following groups: survivors, 174 dyads, in country non-targeted, 150 dyads and returnees, 108 dyads.

Recruitment of participants was based on the following criteria: mothers were required to be Rwandese nationals and residing permanently in the country, a mother with a child of at least 14 years of age residing with her permanently since birth and who voluntarily agreed to participate in the study. Offspring met the study criteria if he or she had lived with his or her mother on a full-time basis until the minimum age of 7 years, agreed to voluntarily participate in the study and was capable of self-reporting. Participants with communication difficulties, who had experienced a mental health crisis at the time of the study, or who had a recent experience of a traumatic event or serious physical illness were excluded. Data were analysed using the Statistical Package for the Social Sciences: (SPSS version 25).

### Socio-demographic characteristics

The study interviewed 432 pairs of mothers and their offspring including 174 survivors, 150 in-country non-targeted, and 108 returnees. The mothers’ mean age was 48.5 (SD = 8.01) while the minimum and maximum age was 32 and 87 years respectively. The majority of mothers (64.12%) were married at the time of the interview while 22.69% were widows. Of the 432 mothers, 82.64% (*n* = 357) reported having completed primary school, 12.96% (*n* = 56) had completed secondary education, and only 0.93% (*n* = 4) had completed university. The Southern Province had the highest representation in the sample with 54.17% (*n* = 234) of participants followed by the Eastern Province with 28.01% (*n* = 121) of the sample. The mean age for the offspring participants, was 17.38 (SD = 2.02) and the minimum and maximum ages were 14 and 22 years respectively. All offspring lived in their respective households their entire lives. Of the 432 offspring participants, 60.88% (*n* = 263) were female and 39.12% (*n* = 169) were male. Furthermore, 65.74% (*n* = 284) of offspring participants had completed secondary school, while 34% (*n* = 147) had completed primary school education. For further details please consult Tables 2 and 3 in Appendix.

### Measures

#### The life events checklist (LEC-5)

The Life Events Checklist (LEC-5) was used to explore traumatic events experienced by the mothers. LEC-5 is a self-report measure designed to screen for potentially traumatic events in a respondent’s lifetime.

The LEC-5 assesses exposure to 16 events known to potentially result in PTSD or distress and includes one additional item assessing any other extraordinarily stressful event not captured in the first 16 items [8].

#### The post-traumatic checklist PCL-5)

The PCL-5 is a 20-item self-report measure that assesses the 20 *DSM-5* symptoms of PTSD. The PCL-5 has a variety of purposes, including: monitoring symptom change during and after treatment, screening individuals for PTSD, and making a provisional PTSD diagnosis. A PCL-5 cut- off point of 33 appears to be a reasonable value to propose until further psychometric work is available [8].

#### The University of California los Angeles Posttraumatic stress disorder reaction index (UCLA-PTSD-RI)

The University of California at Los Angeles’ Posttraumatic Stress Disorder Reaction Index (UCLA-RI) has been used to measure trauma and traumatic events in offspring [9].

The new *DSM-5* version is a semi-structured interview that assesses a child’s trauma history and the full range of *DSM-5* PTSD diagnostic criteria among school-age children and adolescents. UCLA-RI has adequate reliability and validity. A cut-off of 38 has a sensitivity of 0.93 and specificity of 0.87 in detecting PTSD.

## Results

Results revealed 30.2% of our sample met the criteria for significant post-traumatic stress disorder. The prevalence of PTSD by generation is presented in Table 1. The odds of experiencing PTSD were 75% lower for offspring than their mothers (OR = 0.25, *p* < .001); while the likelihood of having PTSD in the sample was associated with the mothers’ survival category (survivors, in country non-targeted, and 1959 returnees). Also, the study found that the odds of having PTSD were 75% lower for the in-country non-targeted group than the survivors’ group (OR = 0.25, *p < .001*), and 70% lower for the returnee group than survivors (0R = 0.30, *p < .001*).

**Table 1:** Prevalence of PTSD in mothers and their offspring

Characteristics	N	%	n(PTSD+)	PTSD%	P_1_	OR	P_2_	95% C. I
General PTSD prevalence	864	100	260	30.2				
Generation								
Mother (base category)	432	50	189	43.8	<.001	1		
Child	432	50	71	16.5		0.25	<.001	[0.18,0.34]
Survival status (child and mother)								
Survivor (base category)	346	40	162	18.8	<.001	1		
In country non-targeted	216	25	53	6.2		0.25	<.001	[0.17,0.34]
1959 returnees	302	35	45	5.2		0.30	<.001	[0.20,0.44]
Generation*Survival								
Offspring*survivor	173	40.3	43	10	<.001	1		
Offspring*In country non-targeted	148	34.5	19	4.4		0.44	0.007	[0.24,0.80]
Offspring* 1959 returnees	108	25.2	9	2.1		0.27	0.001	[0.12,0.59]
Generation*Exposure								
Offspring* mother high exposure	110	25.2	57	51.8	<.001	1		
Offspring*mother moderate exposure	214	49.6	38	18		0.62	0.16	[0.29,1.34]
Offspring * Mother low exposure	108	25.2	40	37.03		1.1	.76	[0.61,2.03]

The likelihood of offspring experiencing PTSD was associated with their mothers’ survival status.

The odds of experiencing PTSD were 66% lower for offspring born from in- country non-targeted mothers than offspring of survivors (OR = 0.44, *p* < .001) and, 73% lower for offspring born from 1959 returnee mothers than offspring survivors (OR = 0.27, *p* < .001). The data indicate that the mother’s status; survivor, returnee or in country non-targeted, influenced their offspring’s development of PTSD, whereas the level or degree of trauma experienced by the mother; high, moderate or low, did not significantly impact the development of PTSD in their offspring.

## Discussion

The results demonstrated that 30 % of our sample met the cut-off for probable PTSD. Mothers were four times more likely to present with PTSD symptoms than their offspring.

Given the horrors of the genocide, it was not remarkable to find mothers who had been exposed to traumatic events and exhibited significant PTSD symptoms. Exposure to multiple traumatic events in adults is associated with high levels of posttraumatic stress disorder, anxiety, and depression [10]. People exposed to multiple traumatic stressors are more likely to develop PTSD and related psychiatric symptoms than people who experience a single exposure to a traumatic event [11]. PTSD affects not only direct victims of trauma, but also their families, observers, as well as direct and indirect participants in traumatic events [12]**.**

Survivor mothers, exhibited greater severity of PTSD symptoms followed by the returnees mothers and lastly, by in country non-targeted mothers. Results of this study demonstrated the likelihood of having PTSD in all sample groups was conditional on the mothers’ survival category (survivors, in country non-targeted, and returnees) with survivors and their offspring being four times more likely to have symptoms than the in-country non-targeted group and three times that of returnee group. PTSD affects not only direct victims of trauma, but also their families, observers, as well as direct and indirect participants in traumatic events.

Studies consistently show that individuals exposed to human-generated traumatic events carry a higher risk of developing Posttraumatic Stress Disorder (PTSD) than those exposed to other kinds of events such as environmental or natural disasters.

The literature reports perceptions of social support before and after a traumatic event as an important factor in determining vulnerability in the development of PTSD [13].The Genocide against the Tutsi was a human-generated traumatic event with catastrophic and far-reaching effects on the entire society. Within 3 months, more than one million Tutsi were killed. The genocide caused far-reaching physical and psychological consequences as a result of the horrific, macabre acts of the killers. Hundreds of thousands of survivors were wounded or permanently physically disabled; widowed, contracted HIV/AIDS, or were forced to live without shelter.

The very fiber of Rwandan society and sense of social cohesion were decimated. The genocide destroyed the mutual trust and sense of unity Rwandans had developed and relied upon for centuries; People separated from one another based on politically reinforced fear, hatred and mistrust [14].

Although most Rwandese have been exposed to traumatic events in their lifetime, the symptoms of PTSD were exacerbated in genocide survivors as a result of their lived experience and chronic exposure to multiple traumas during the genocide.

A curious exception was the returnee group who were not physically present in the country during the genocide, but who experienced the second highest rate of PTSD prevalence.

This indicates that the returnee group was indirectly targeted and affected by the loss of many family members.

Clinical observation and empirical research have shown that the consequences of traumatic events are not limited to the persons immediately exposed to the event, and often significant others in their environment such as children, family members, friends, and caregivers are affected. Children often feel the greatest impact of a parent’s PTSD because of the parent’s lack of ability to function effectively as a parent, due to their PTSD symptoms [15]. Changes in parental functioning can lead to unmet family needs and increased levels of stress for the family, specifically for the children [16, 17].

Our study demonstrated the likelihood of children having PTSD was conditional upon their mothers’ survival status with offspring born from survivor parents twice as likely to manifest PTSD symptoms as offspring of in-country non-targeted mothers and at least four times that of offspring of the returnees.

Parents suffering from PTSD may have observable difficulties in their relationships with their spouse and children. Withdrawal, isolation, inability to express emotions, overprotection and over-control of their children are challenges they encounter while trying to function within the family system. In such family environments, important developmental stages are potentially disrupted; such as bonding, attachment and individuation, due to the fact that the parent’s struggle with mental health issues places the child in an atmosphere charged with high levels of anxiety, depression and impulsivity. Parental engagement in violence against children and neglect may expose children to increased trauma. The more victims of a traumatic event manifest symptoms of PTSD, the more likely their children are also at risk of suffering from PTSD [11]. It is worth noting that the returnee mothers had the second highest level of PTSD symptoms while their offspring had fewer symptoms and the difference between symptoms of offspring born from survivor mothers and in country, non-targeted mothers was insignificant.

Further studies are needed to explore protective factors for children born from returnee mothers and to determine risk factors for the other two groups.

Our data indicate the probability of offspring having PTSD was not a condition of their mother’s trauma exposure. The literature reports when the traumatic experiences of parents are not addressed, their emotional and behavioral legacy is often passed down to their children. Interestingly, this was not the case for our sample. We found no significant relationship between the mother’s level of trauma and their offspring’s development of PTSD.

## Conclusion

Results of this study are consistent with the findings of previous research on the prevalence of PTSD symptomatology in the Rwandan population [13].

The current study provides an important addition to the literature as it explores PTSD among three groups of mothers categorized by their levels of exposure to the 1994 genocide against Tutsi.

A very high prevalence rate of PTSD was found among survivor mothers, followed by returnee mothers and lastly, by in country non-targeted mothers. These results serve to further strengthen current national efforts to heal PTSD, focusing on the genocide survivors. Overall, the results reveal a high prevalence of PTSD in Rwandese, and the most vulnerable group is the survivors and their offspring. Efforts to delineate the psychosocial, genetic or narrative factors contributing to PTSD in Rwandan offspring will be very important in further research studies.

## Limitations

Parent participants in this study were restricted to biological mothers of their offspring as they are characteristically the child’s principal attachment figure [4]. Further studies are needed to explore the impact of both parents on the development of PTSD in offspring as they can become attached to adults other than the primary caretaker who are sensitive and responsive in social interpersonal interactions [5].

Differences of prevalence of PTSD among the three groups of offspring were identified but further research is needed to explore possible factors contributing to the development of PTSD in offspring of mothers in the three identified groups.

Prevalence of PTSD in the mother-offspring dyads across the three groups was explored in eight out of thirty districts in Rwanda. A larger study covering the entire country would be necessary to verify the generalizability of the present study findings on the entire Rwandan population. As present districts were selected based on the criteria of having large populations of survivors and those who lived outside the country during the 1994 Genocide against the Tutsi, it would interesting to find out what results would occur from a random selection of districts.

## Data Availability

The data sets used and/or analyzed for this study are available from the corresponding author upon reasonable request.

## Appendix

**Table 2:** Characteristics of mother participants

Characteristics		n	Percentage
Survival status	Survivors	174	40.28
	In country non targeted	150	34.72
	1959 returnees	108	25.00
Age group	32–42	71	16.44
	43–54	201	46.53
	55–64	148	34.26
	65+	12	2.78
Marital status	Married	277	64.12
	Divorced	49	11.34
	Separated	8	1.85
	Widow	98	22.69
Education	Primary	357	82.64
	Secondary	56	12.96
	TVET	13	3.01
	University	4	0.93
	Adult literacy	2	0.46
Province of residence	Kigali	41	9.49
	South	234	54.17
	West	36	8.33
	East	121	28.01

**Table 3:** Characteristics of offspring participants

Characteristics		n	Percentage
Survival status	Survivors	174	40.28
	In country non targeted	150	34.72
	1959 returnees	108	25.00
Age group	14–16	156	36.11
	17–19	202	46.76
	20–22	74	17.13
Sex	Male	169	39.12
	Female	263	60.88
Education	Primary	147	34.03
	Secondary	284	65.74
	TVET	1	0.23
Province of residence	Kigali	40	9.26
	South	223	51.62
	West	36	8.33
	East	133	30.79

## Abbreviations

DSM: Diagnostic Statistical Manual
LEC-5: Life Events questionnaire DSM-5
NISR: National Institute of Statistics
OR: Odds Ratio
PCL-5: PTSD Checklist for DSM-5
PTSD: Post Traumatic Stress Disorders
SPSS: Statistical Package for the Social Science
UCLA-PTSD-RI: University of California at Los Angeles, Posttraumatic Stress Disorders Reaction Index for DSM-5
